# Insight Into Trophic Niche Differentiation in *Labeobarbus* (Cyprinidae) in the Luhoho Basin (Upper Congo Basin)

**DOI:** 10.1002/ece3.71171

**Published:** 2025-04-03

**Authors:** Tchalondawa Kisekelwa, Wilondja Alimasi, Mudagi Joyeuse, Musombwa Kubota, Heri Muzungu, Archimède Mushagalusa Mulega, Pieter Lemmens, Emmanuel Vreven, Jos Snoeks, Mulungula Masilya, Steven Bouillon, Benjamin Lejeune

**Affiliations:** ^1^ Centre for Research in Biodiversity, Ecology, Evolution and Conservation (CRBEC) Bukavu Democratic Republic of the Congo; ^2^ Unité d'Enseignement et de Recherche en Hydrobiologie Appliquée (UERHA), Biology Department Institut Supérieur Pédagogique de Bukavu (ISP) Bukavu Democratic Republic of the Congo; ^3^ Département de Biologie Centre de Recherche en Hydrobiologie (CRH) d'Uvira Uvira Democratic Republic of the Congo; ^4^ Laboratory of Aquatic Ecology, Evolution & Conservation, Biology Department KU Leuven Leuven Belgium; ^5^ Leibniz Institute für Gewasserökologie Und Binnenfischerei (IGB) Berlin Germany; ^6^ Vertebrates Section, Ichthyology Royal Museum for Central Africa Tervuren Belgium; ^7^ South African Institute for Aquatic Biodiversity Grahamstown South Africa; ^8^ Fish Diversity and Conservation, Biology Department KU Leuven Leuven Belgium; ^9^ Department of Earth and Environmental Sciences KU Leuven Leuven Belgium; ^10^ Laboratory of Ecology and Conservation of Amphibians (LECA), Freshwater and OCeanic Science Unit of reSearch (FOCUS) University of Liège Liège Belgium

**Keywords:** freshwater fish ecology, isotopic niche, mouth morphology, niche differentiation, trait divergence, trophic lineage diversification

## Abstract

Mouth morphology plays a crucial role in determining the trophic ecology of fish and sometimes underpins important lineage diversification. Freshwater teleost fish species belonging to the genus *Labeobarbus*, commonly found in Africa, exhibit intra‐ and interspecific variation and differences in the lower jaw occurring within and between species, respectively. Different phenotypes include a curved U‐shape (‘rubberlips’), a straight lower jaw (‘chiselmouth’) and an intermediate morphology known as the smiling phenotype. In some cases, smiling originates from hybridisation between chiselmouth and rubberlips. However, the trophic relationships of different mouth morphologies in the *Labeobarbus* taxa are still not well understood, particularly in the Congo Basin. Understanding the trophic ecology of *Labeobarbus* can enhance understanding of adaptive processes in morphologically diverse lineages. This study aims to investigate how differences in mouth morphology among multiple *Labeobarbus* species in the Luhoho River (Upper Congo Basin) link with different trophic niche uses. We combined information from gut morphometry, gut contents and stable isotope analyses on 202 fish specimens representing six species across four tributaries of the Middle Luhoho. All approaches consistently revealed trophic niche partitioning between chiselmouth and rubberlip species, respectively, more herbivorous/detritivorous and more insectivorous on the omnivory spectrum. In addition, trophic differences were also found between species within each mouth phenotype. Interestingly, the trophic niche of the smiling phenotype differed strongly from those of other phenotypes at all sites except for *L. paucisquamatus*, for which the trophic niches overlapped in Tchinganda. The pattern of trophic niche of *Labeobarbus* suggests subtle strategies to partition feeding resources when they occur across a narrow hydrographic scale.

## Graphical abstract

Six *Labeobarbus* species occur in the Middle Luhoho, a left‐bank affluent of the Lowa River, including two chiselmouth species *(Labeobarbus brauni*, *L. longidorsalis*) and three rubberlip species (*L. caudovittatus*, *L. longifilis*, *L. paucisquamatus*), as well as an intermediate phenotype referred to as the smiling phenotype. Based on intestinal content and stable isotope approaches, chiselmouth species were found to feed on periphyton (algae, moss, and detritus), while rubberlip species were revealed to feed on insects. Isotopic niche modelling indicated that each species has achieved its isotopic niche differentiation. The intermediate phenotype tends to exhibit an intermediate value in its isotopic data. Nevertheless, the isotopic niches of *L. paucisquamatus* and intermediate (smiling) phenotypes overlapped, denoting that they could partially feed on the same food types.

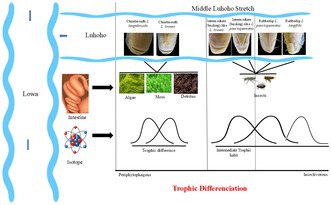

## 1 Introduction

In aquatic animals such as fish, the morphology of the mouth (Bonato et al. 2017) and the digestive apparatus (Moyle and Cech 2004) are often tightly associated with their feeding mode, food types and habitat use (Smith and Skúlason 1996; de Graaf et al. 2010; Paugy and Lévêque 2017). For example, differences in jaw teeth shape are associated with different herbivorous diets in African cichlid fishes, specialising either on filamentous algae (using the anterior row of tricuspid teeth to nip and tear filamentous algae from rocks) or on unicellular algae (relying on a high density of bicuspid teeth to comb unicellular algae from filamentous ones attached to rocks) (Yamaoka 1983). Longer guts with a greater surface area typify species that feed on detritus and algae and that may take in high proportions of indigestible materials such as sand, mud or cellulose, whereas carnivorous species tend to have shorter guts (Moyle and Cech 2004; Paugy and Lévêque 2017). Studying the links between morphological variation in trophic structures (e.g., mouth or gut morphology) and differences in resource use across species can inform about the potential processes promoting phenotypic or species diversification.

*Labeobarbus* is a genus of teleost fish of the family Cyprinidae widely distributed across multiple aquatic systems in Africa (Vreven et al. 2016). The genus is composed of a particularly diverse set of species with divergent morphological traits in mouth parts, both at the intra‐specific and inter‐specific levels (Nagelkerke et al. 1994; de Graaf et al. 2010; Vreven et al. 2016; Levin et al. 2019; Kisekelwa et al. 2020). In some cases, mouthpart divergence does not necessarily imply further body transformations (e.g., Kisekelwa et al. 2020; Katemo et al. 2023). Multiple *Labeobarbus* species can be found in syntopy, sometimes representing highly morphologically diverse assemblages of species, such as in Lake Tana in Ethiopia (Nagelkerke et al. 1994; Nagelkerke and Sibbing 1998; de Graaf et al. 2008).

**FIGURE 1:**
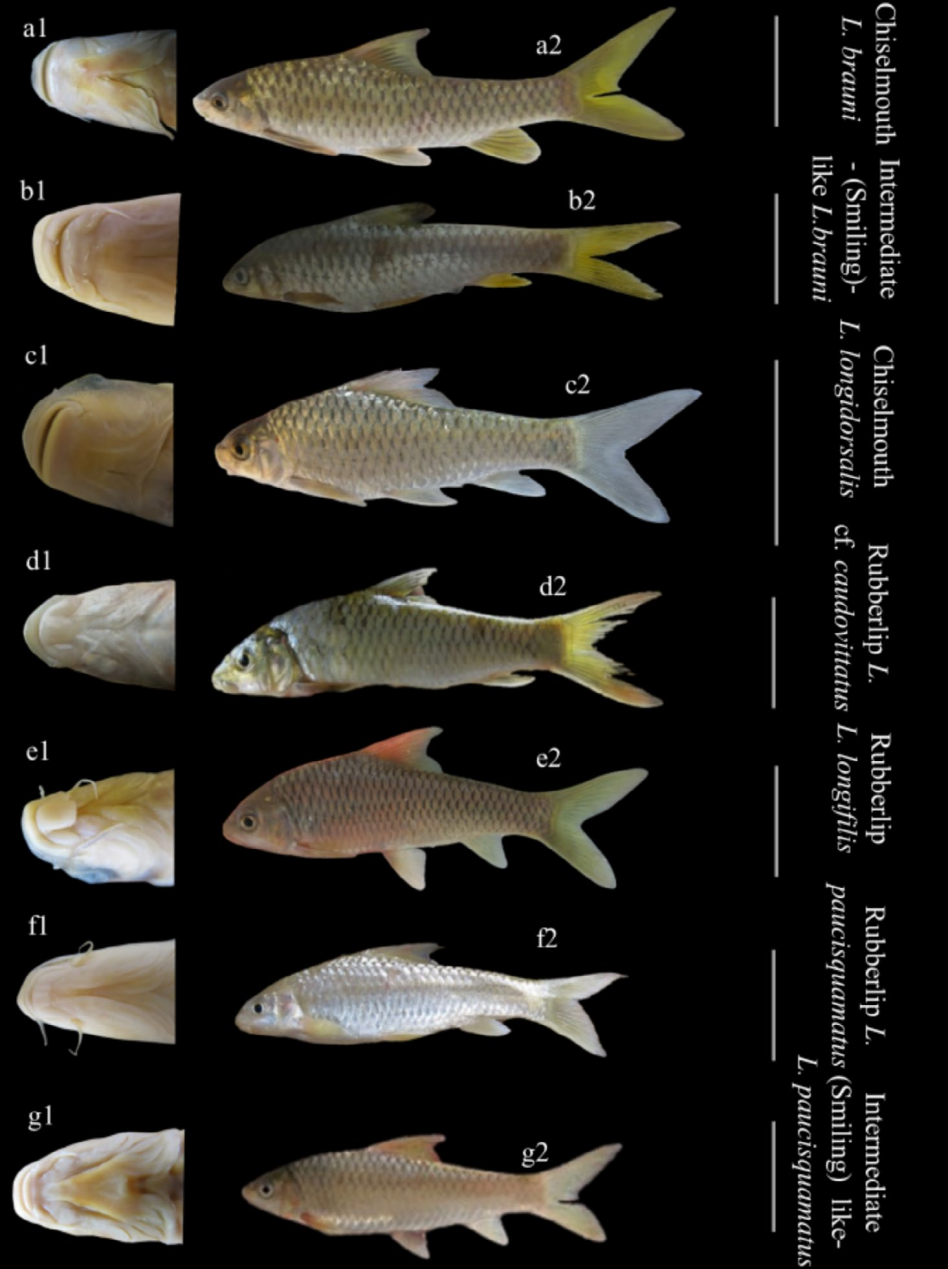
Mouth morphology (1) and habitus (2) of *Labeobarbus* species from the Middle Luhoho. The three first pictures belong globally to chiselmouth phenotype having a keratinised cutting edge on their lower jaw. This phenotype is represented by (a) *L. brauni* (Pellegrin, 1935); (b) A morphotype of *L. brauni* having an intermediate morphology dubbed smiling specimen with a sharp keratinised cutting edge but, with fleshy mouth corners and (c) *L. longidorsalis* (Pellegrin, 1935). The remaining pictures belong to rubberlip species and an intermediate morphotype. (d) *Labeobarbus* cf. *caudovittatus* (Boulenger, 1902) and (e) *L. longifilis* (Pellegrin, 1935); both have a well‐developed mental lobe. (f) *L. paucisquamatus* (Pellegrin, 1935) bears a poorly‐developed, free fleshy mental lobe. (g) Intermediate (smiling) phenotype like *L. paucisquamatus*, also regarded as smiling phenotype, lacking a mental lobe on the lower jaw. Size: 100 ≤ 200 mm SL.

There are two major phenotypes based on mouth morphology in *Labeobarbus*: ‘chiselmouth’ (Chis) (Figure 1a,c), referring to the species which have a keratinised cutting edge on the lower jaw, and ‘rubberlips’ (Rub), that is, lobed mouth, referring to species with a well‐developed mental lobe (Figure 1d–f) (Vreven et al. 2016). In some areas, intermediate morphologies between the two phenotypes occur as well. Indeed, some of the intermediate phenotypes have a keratinised cutting edge on the lower jaw coupled to either a minute, attached mental lobe or lacking it completely, such as the ‘smiling’ phenotype (see Levin et al. 2019, 2021) (Figure 1b,g). Others have a U‐shaped lower jaw across which the median section is still keratinised, with developed lips at the extremity. These specimens are considered intermediate as they cannot be categorised into one of the major mouth phenotypes. The specimens belonging to the aforementioned phenotypes are reported in the Luhoho Basin, across the Middle stretch, a left‐bank tributary of the Lowa Basin (Upper Congo) (Kisekelwa 2019; Kisekelwa et al. 2020). However, the origin of polymorphism of the mouth in *Labeobarbus* is not yet well resolved.

Prior studies revealed that the mouth variation could result from adaptation to particular environmental conditions (Groenewald 1958; Crass 1964; Gaigher 1975) or from hybridisation between *L. longifilis* (Rub) and *L. brauni* (Chi) (Kisekelwa 2019). Differences in mouth structure can influence the feeding behaviour of *Labeobarbus* species, irrespective of their origin. Indeed, trophic differentiation has been observed between chiselmouth species (Levin et al. 2021); while, it can rarely occur between hypertrophied and normally developed lips ectomorphs in the river systems in Ethiopia (Levin et al. 2023). However, few data are available that document the extent to which *Labeobarbus* species use different trophic resources either within or among mouth phenotypes in river systems in the Congo Basin. The *Labeobarbus* taxa occurring in the Middle Luhoho provide an opportunity to examine the ecological implications of morphological differences in *Labeobarbus* by exploring how multiple species use and share resources between populations in different environmental settings. Chiselmouth species such as *L. brauni* (Chi) and *L. longidorsalis* (Chi) may be more herbivorous, following Levin et al. (2021), but intra‐morph differences are also expected according to differences in habitat use observed among species (Kisekelwa et al. 2021). By contrast, one may expect rubberlip species to feed mainly on macrophytes, insect larvae and detritus (Nagelkerke and Sibbing 1998; de Graaf et al. 2008) or to be invertivorous or omnivorous, following Levin et al. (2023). The smiling phenotype can be more detritivorous with the occurrence of insects, following Levin et al. (2019).

This study aimed to identify the diet composition, explore the isotopic niche of each *Labeobarbus* species in the Middle Luhoho (Lowa, Upper Congo Basin), and evaluate whether this isotopic niche remains consistent across rivers and/or when different species with the same or different mouth phenotype(s) occur in sympatry or not.

## 2 Material and Methods

### 2.1 Study Area and Sampling

**FIGURE 2:**
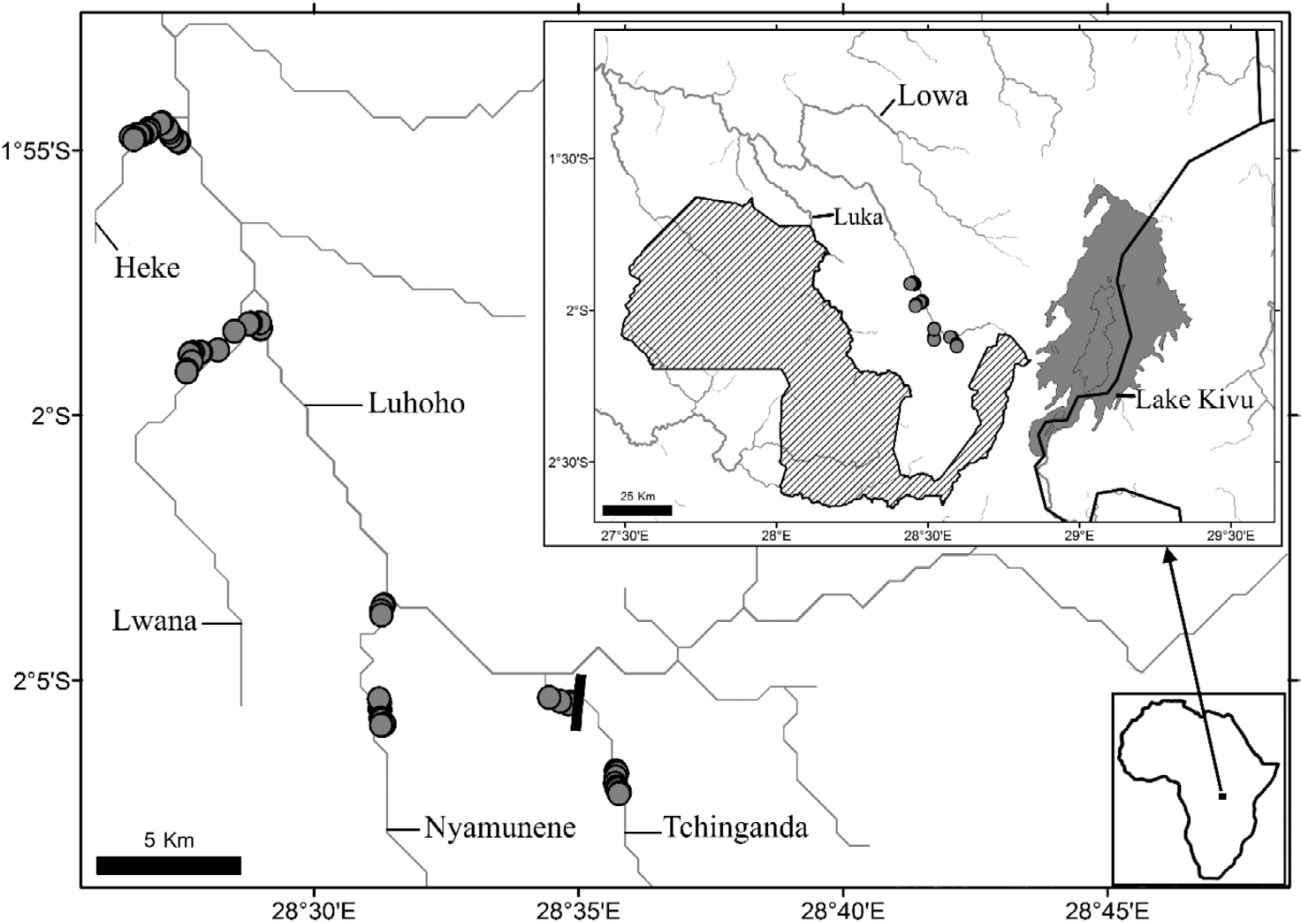
The sampling sites in the Middle Luhoho used in Kisekelwa et al. (2021). Striped area represents the Kahuzi‐Biega National Park; and▐: Tchinganda Falls at Bulambika.

Sampling was conducted in four left‐bank tributaries of the Luhoho River, including the Tchinganda, Nyamunene, Lwana and Heke (downstream order; Figure 2). The Luhoho River drains the Kahuzi and the Biega mountains and east‐northward across *ca* 25–50 km from the Kahuzi Biega National Park (KBNP) border. The Luhoho Basin belongs to the left‐bank tributaries of the Lowa [Middle Lowa, following Kisekelwa et al. (2020)], which, in turn, is a right‐bank tributary of the Upper Congo (Lualaba). Brown and Abell (2005) included the Luhoho River and part of the Lowa system, which drains the western mountains of the Albertine Rift Valley of East Africa, in the Albertine Highland Ecoregion. The ecoregion belongs to a tropical and wet climate (Brown and Abell 2005). The dry season occurs from June–July, while the heavy rainy season is from late October to December (observation made by authors).

The catchment of these rivers is populated by an intermediate forest along the Tchinganda catchment from about 800 to 1000 m asl and by a typical equatorial rain forest at about 700 m asl, representing the landscape of the remaining rivers. The entire catchment of the Tchinganda River is almost denuded due to intensive agriculture. In contrast, only some stretches of the remaining rivers have apparently been impacted. In the Nyamunene catchment, only the lower section is strongly affected by human activities, including agriculture, palm oil production and artisanal mining. The Lwana and Heke River catchments are mainly covered by dense forest but also host palm plantations and an introduced plant species, *Bellucia aubletii* Naudin, 1850. Traditional oil production plants are used and set along the zone adjacent to the bank of the river system. This activity has been recognised as the main human activity impacting the river banks in the Luhoho Basin (Brown and Abell 2005).

The data were collected during two successive dry seasons (2015 and 2016). Fish were sampled following Kisekelwa et al. (2021) using a cast net during the daytime. Live *Labeobarbus* specimens from each catch were euthanised by MS222 or clove oil before identification using the European Directive 2010/63/EU (Kisekelwa et al. 2021). A total of 202 specimens were collected over the sampled rivers. Empty guts were excluded in calculating the frequency occurrence index (see below), while low sample sizes were excluded from statistical tests. The sample number of discarded specimens is calculated from the total number of samples used to investigate the trophic part (gut content approach) of this study (Table 1). Specimens were identified to the species level and attributed to three morphotypes: Chiselmouth, which includes *L. brauni* (Chi) and *L. longidorsalis* (Chi); Rubberlips, represented by *L. caudovittatus* (Rub), *L. longifilis* (Rub) and *L. paucisquamatus* (Rub) and smiling phenotype.

**TABLE 1:** Overview of the number of specimens per species and sampled rivers included in this study.

	Tchinganda	Nyamunene	Lwana	Heke	Luhoho	Total
*L. brauni* (Chis)	9/4	23/9	3/2	25/15	0/0	60/30
*L. longidorsalis* (Chis)	0/0	0/0	10/9	1/1	0/0	11/10
*L. caudovittatus* (Rub)	0/0	1/0	2/1	3/3	0/0	6/4
*L. longifilis* (Rub)	0/0	8/1	11/11	17/11	27/11	63/34
*L. paucisquamatus* (Rub)	19/9	9/7	1/0	1/1	1/1	31/18
Smiling (Int)	10/8	0/0	1/2	2/1	0/0	14/11
**Total**						**185/107**

*Note:* In the last column, the numerator refers to the number of specimens included in the intestine content approach, and the denominator represents the samples used for stable isotope analyses. Chis, Rub and Int refer to Chiselmouth, Rubberlip and intermediate mouth phenotypes respectively.

A dorsal muscle sample was taken on the right side of each specimen together with the entire digestive tract (intestine). Putative food items were sampled in each location (including leaves, fruits, shellfish and insects). Muscle and putative food items were dried in the field by exposing the samples to the sun while avoiding flies from laying eggs on the samples. Each digestive tract was kept in a cryovial tube of 30 mL or 50 mL in 4% formaldehyde. Morphometric parameters were measured from each specimen, including the standard length (SL in mm) and the mouth width (mm). The intestine length was measured before gut content observation. The prognathous species *Labeobarbus* sp. ‘isangi’ was not included in this study as it rarely occurs in the Middle Luhoho and was absent from our catches during the ecological sampling carried out in the basin.

### 2.2 Trophic Analysis

Three complementary methods, notably the morphometry related to gut, gut content and Stable Isotope Analysis, were used to explore the trophic characteristics of *Labeobarbus* taxa in the Luhoho. Gut content analysis provides a snapshot of the diet but may overlook quickly digested items and temporal variation in food assimilation (Hyslop 1980; Elser et al. 2000; Pasquaud et al. 2007). Stable isotope analysis offers a more integrated picture (Jackson et al. 2011; Newsome et al. 2007) but, can be uncertain for generalist or omnivorous consumers (Davis et al. 2012). Additionally, analysing morphological parts, such as mouth width (Cohen et al. 1993; Wilson 1975) and intestine length in fish, can provide valuable insights into dietary habits (Paugy and Lévêque 2017; Levin et al. 2019).

### 2.3 Analysis of Morphological Parameters

Gut and mouth size were examined to explore how species with the same or different mouth anatomy can differ in their mouth size. Gut morphology was investigated by calculating the Intestinal coefficient (Ci) for each specimen separately as the intestine length divided by the standard length (SL) of each specimen (Paugy 1994). Subsequently, the mouth width and intestine length of species within and between pairs of mouth phenotypes were compared using ANOVA I (Tukey tests) and the Kruskal Wallis (Mann Whitney U tests), respectively. The alpha level was adjusted using the Bonferroni correction in the default menu of Past software version 3. Subsequent tests were performed on specimens corresponding to the same range of sizes between species after obtaining a *p* value > 0.5 (Vranken et al. 2019; Kisekelwa et al. 2022). *Labeobarbus brauni* (Chi) [*n* = 13 (89.4–112.9 mm)] and *L. longidorsalis* (Chi) [*n* = 7 (88.3–112.4 mm)] and between the former [*n* = 5 (96.7–105.1 mm)] and smiling phenotype [*n* = 5 (96.6–109.0 mm)] as well as between *L. brauni* (Chi) [*n* = 47 (89.4–177.6 mm)] and *L. longifilis* (Rub) [*n* = 47 (80.4–179.0 mm)], and between the former and *L. paucisquamatus* (Rub) [*n* = 37 (81.3–175.3 mm)].

### 2.4 Gut Content Analysis

Gut content analyses were performed on 185 samples belonging to rubberlips (see caption of Figure 1), chiselmouth (see caption of Figure 1) and smiling (see caption of Figure 1) phenotypes (Table 1) to characterise the food item composition and assess the foraging behaviours of *Labeobarbus* species in the Middle Luhoho directly. Because cyprinids lack a specialised sphincter and thus lack a true stomach (Helfman et al. 2009), the entire intestine for each specimen was analysed following Pasquaud et al. (2007). Items were sorted out and identified to the most precise rank possible using a stereoscopic microscope magnifying up to 60 times (Nikon Model C‐PS). Insects were generally crushed, preventing any further direct taxonomical identification or precise count (see Hyslop 1980), but in some cases, we were able to obtain more precise taxonomic information by associating particular body parts such as legs or head fragments with insects sampled in the local environment (see discussion).

The diet of each species was described using two standard dietary indices, namely the frequency of occurrence (%Fo) and the volumetric index (%Vi) of each food category (see Hyslop 1980; Masilya et al. 2011, 2020). The volume of each food category was assessed through the point approach, i.e., by squashing the content on a plate and attributing a surface covered by each food category (Hyslop 1980). The volumetric index gives a relative volume of each prey upon the total volume of each intestine. It can be extended as an indication of the biomass that each prey brings into the diet composition of a studied species. The percentage of Lauzanne's dietary index (%*IAi*) (Lauzanne 1976) has been computed for each food category *i* to compare diets for each species: %*IA*_*i*_ = $\frac{{\mathrm{IA}}_{i}}{\sum_{i=1}^{n}{\mathrm{IA}}_{i}}\times 100$ with

$${\mathrm{IA}}_{i}=\left(\%{\mathrm{FO}}_{i}\times \%V_{i}\right)/100.$$

Generalised Linear Models (GLMs) with a binomial distribution (Zuur et al. 2009) were used to assess the effects of size (SL), mouth width (MW) and intestine length (IL) on the volumetric index of the two main food categories: ‘insects’ and the combined ‘detritus‐algae‐moss’. Such an approach is appropriate to search for explanatory variables' effects on diet variation (Belleggia et al. 2012). The values of SL, MW and IL were all log‐transformed, while the volumetric index of each food category was squared root transformed to reduce skewness (Pereira et al. 2007; Kisekelwa et al. 2021). The results of the test for which one variable only was significant were presented.

Analysis of similarities (ANOSIM) (Clarke 1993) was performed on the volumetric index matrix to test for significant differences between the diets of the different chiselmouth and rubberlip species (global test followed by pairwise tests between each species pair). The *r* value provided by ANOSIM indicates the degree of dissimilarity between diets, where *r* = 0 indicates the absence of difference and *r* = 1 indicates a complete separation of the trophic niches based on diet composition. Whenever two species had significantly different diets according to ANOSIM, we subsequently calculated the contribution of each prey type to the observed global diet difference using similarity percentage (SIMPER) analysis (Clarke 1993). We used GLM and ANOSIM functions from the vegan package for these statistical analyses (Oksanen et al. 2016) of R version 3.6.1 (R Core Team 2023).

### 2.5 Stable Isotope Analysis: Raw Data Exploration and Isotopic Niche Modelling

The Stable Isotope Analysis allowed for comparing isotopic niches between *Labeobarbus* species and provided initial insights into the dietary habits of these *Labeobarbus* in sympatry across the river affluents of the Middle Luhoho. The isotope dataset comprised 107 *Labeobarbus* samples and 24 samples representing putative food resources collected across the stretches of five sampled rivers. These putative food samples are represented by benthic mosses, Pteridophyta, leaves, plant seeds, crabs and insects, each containing a number of replicates. Samples were dried in the field and laboratory in an oven at 60°C for 48 h. Crab samples were acidified in 10 μL of HCl 10% to eliminate all inorganic carbon in the shell. Samples were homogenised into a powder using mortar and pestle, with the addition of liquid nitrogen for vegetation samples. A maximum of 1 mg per sample was individually loaded in Ag or Sn cups for isotopic analysis. Samples were analysed on a Thermo Flash HT/EA or EA1100 coupled to a Thermo Delta V Advantage isotope ratio mass spectrometer. We used a combination of IAEA‐600 (caffeine) and two in‐house standards (Leucine and tuna muscle tissue) which were previously calibrated versus certified standards, to correct δ^13^C and δ^15^N data. Reproducibility of δ^13^C and δ^15^N measurements was typically better than 0.15‰.

A simple biplot between δ^13^C and δ^15^N was generated to explore the isotopic niche use among species (a proxy for a trophic niche). Bivariate standard ellipses representing core isotopic niches of each *Labeobarbus* species were generated using the package SIBER version 2.1.0 in R version 3.3.1 (Jackson et al. 2011). Areas of the ellipses associated with each group (Standard Ellipse Area B; SEA_B_) were computed using Bayesian inference (MCMC parameters: 2 chains, 200,000 iterations, 10,000 burn‐ins, thins = 50, and using an inverted Wishart prior) to account for sample size differences among groups. SEA_B_ were compared among the different species using direct pairwise comparisons of their posterior distributions. The percentage of shared niche space (SNS) was calculated between each species’ niche based on single estimates of standard ellipses area corrected for small sample sizes (SEAc). SNS between species ‘a’ and ‘b’ was calculated following the equation: SNS = 100 * Overlap(ab)/(SEAc(a) + SEAc(b) − Overlap(ab)) (Lejeune et al. 2021). Segregation among the different *Labeobarbus* species niches at the global scale (data from all sites combined) was further assessed by comparing their δ^13^C and δ^15^N values, respectively used as proxies for basal resource use and trophic position of fish. We tested for differences in δ^13^C and δ^15^N values among *Labeobarbus* species by conducting pairwise PERMANOVA tests (Euclidean distances, 9999 unrestricted permutations of the residuals and Type‐III sums of squares) (Anderson 2001) using PRIMER version 7 software (Clarke and Gorley 2006) and the PERMANOVA+ add‐in (Anderson et al. 2008). *p* values were adjusted for multiple testing using Bonferroni correction.

## 3 Results

### 3.1 Gut Morphology of *Labeobarbus* Taxa

Great changes were observed in intestine length both within and among mouth phenotypes (Table 2). Mouth width and intestine length were significantly different only between *L. brauni* (Chi) and *L. longidorsalis* (Chi); between *L. brauni* (Chi) and both *L. longifilis* (Rub) and *L. paucisquamatus* (Rub). Furthermore, *L. brauni* (Chi) and the smiling phenotype were significantly different in mouth width (*p* < 0.001). After removing the allometric effect (see Materials and Methods) and testing the MW data on subsets for which the average SL was similar (with *p* > 0.5), MW and IL between *L. brauni* (Chi) and *L. longidorsalis* (Chi) did not differ significantly (*p* > 0.05). Inversely, MW between *L. brauni* (Chi) and smiling differed significantly (*p* < 0.05) and *L. brauni* (Chi) consistently differed significantly for MW and IL from *L. longifilis* (Rub) and *L. paucisquamatus* (Rub) (*p* < 0.001). Generally, *L. brauni* (Chi) has a large mouth and a longer intestine than rubberlip species. Intestinal coefficients depicting the ratio of intestine length over standard length reflected the variation of the intestine size between *Labeobarbus* species. Indeed, *L. brauni* (Chi) had the highest ratios; *L. longifilis* (Rub) revealed having the lowest ratios, with the three others being intermediate for *L. longidorsalis* (Chi), for *L. paucisquamatus* (Rub) and for the smiling phenotype.

**TABLE 2:** Samples (*N*), size range, mouth width and intestine coefficient obtained for *Labeobarbus* species from the Middle Luhoho.

Species	Morphological parameters				
	*N*	Size range (mm)	Mouth width (median) (mm)	Intestine range (median) (mm)	Intestinal Coefficient
*L. brauni* (Chis)	60.0	76.4–224.9	4.4–20.4 (11.1)	200–1160 (589.1)	1.8–6.7 (15.1)
*L. longidorsalis* (Chis)	11.0	68.9–112.4	4.7–9.9 (6.8)	130–520 (212.5)	1.7–4.7
*L. caudovittatus* (Rub)	4.0	115.4–150.0	8.3–11.9 (10.0)	250–900 (350.0)	2.1–6.9
*L. longifilis* (Rub)	63.0	66.7–257.1	3.8–18.3 (6.4)	60–510 (205.0)	0.7–3.4
*L. paucisquamatus* (Rub)	39.0	74.1–390.0	3.7–12.7 (6.5)	90–790 (226.1)	1.0–5.1
Smiling (Int)	14.0	98.3–175.4	4.3–9.3 (5.7)	125–560 (240.0)	1.2–4.2

*Note:* The number in brackets indicates an outlier value of the intestinal coefficient for an individual. Chis, Rub and Int refer to chiselmouth, rubberlip and intermediate mouth phenotypes, respectively.

### 3.2 Gut Contents

**FIGURE 3:**
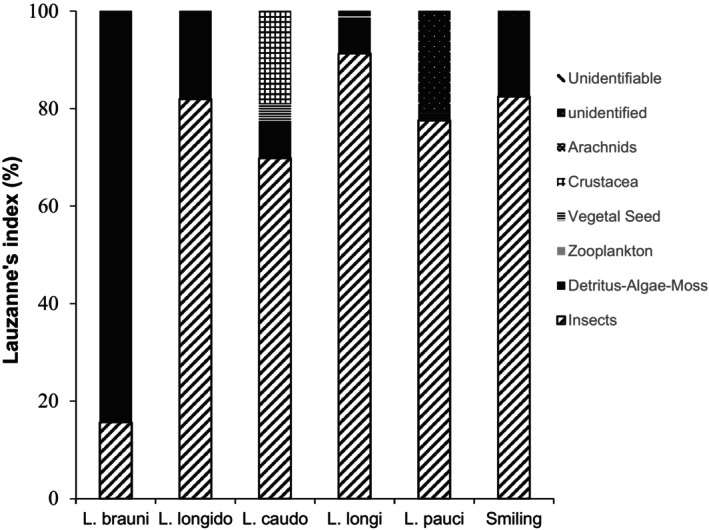
Lauzanne's index of food categories consumed by *Labeobarbus* species in the Luhoho Basin. *Labeobarbus longido* (*L. longidorsalis*), *L. caudo* (*L. caudovittatus*), *L. longifi* (*L. longifilis*), *L. pauci* (*L. paucisquamatus*). Unidentifiable items indicate contents that have undergone advanced digestion, while unidentified items are recognisable in form but cannot be named.

*Labeobarbus* species displayed a wide‐ranging diet that could be grouped into six categories: insects, detritus‐algae‐moss, zooplankton, seeds, crabs and arachnids (Table 3 and Figure 3). Insects were the most frequent food type across all phenotypes and species, with a frequency of occurrence (%FO) reaching 100% in *L. caudovittatus* (Rub), *L. longidorsalis* (Chi), and the smiling phenotype, while they represented 86.2% and 95.2% in the two rubberlip species, *L. paucisquamatus* (Rub) and *L. longifilis* (Rub), respectively. In *L. brauni* (Chi), insects represented 62.2% of the diet in terms of occurrence (%FO) and 18.5% in terms of volume, whereas detritus‐algae‐moss were much higher both in %FO (92.9%) and volume (67.1%). In *L. longidorsalis* (Chi), another chiselmouth species, detritus‐algae‐moss had a %FO of about 55.5% but represented only 17.8% of the diet in terms of volume, whereas insects represented 44.8% in terms of volume. In the rubberlip phenotype, *L. caudovittatus* (Rub), *L. longifilis* (Rub) and *L. paucisquamatus* (Rub), insects were the major prey, representing 36.2%, 50.1% and 39.2% of the overall gut contents' volume, respectively. Items from plant origin, seeds, zooplankton, crabs and arachnids were seldom found in rubberlips. In the smiling phenotype, the %FO of detritus‐algae‐moss was 53.8%, with a volume of 22.5%. In the smiling phenotype, insects occupied an important volume corresponding to 60.0% of the total gut content volume.

**TABLE 3:** Contribution of food items expressed as frequency occurrence (Fo) (%), volumetric index (Vi) (%) in gut contents of *Labeobarbus* spp. from the Middle Luhoho.

Food items	Fo (%)						Vi (%)					
	*L. brau*	*L. longid*	*L. caudov*	*L. longif*	*L. pauci*	Smiling	*L. brau*	*L. longid*	*L. caudov*	*L. longif*	*L. pauci*	Similing
Insects	62.2	100.0	100.0	95.2	86.2	100.0	18.5	44.8	36.2	50.1	39.2	60.0
Detritus‐Algae‐Moss	92.9	55.5	33.3	35.9	13.7	53.8	67.1	17.8	11.7	10.4	4.3	22.5
Zooplankton	3.44	0.0	0.0	3.1	3.4	0.0	0.0	0.0	0	0.1	0.1	0.0
Vegetal Seed	0.0	0.0	16.6	12.6	3.4	0.0	0.0	0.0	11.7	2.9	1.4	0.0
Crustacea	0.0	0.0	33.3	1.5	3.4	0.0	0.0	0.0	29.4	0.3	3.6	0.0
Arachnids	0.0	0.0	0.0	3.1	3.4	0.0	0	0.0	0	0.2	0.4	0.0
Unidentified	1.7	0.0	0.0	9.5	62.0	23.1	0.2	0.0	0	4.4	14.5	2.8
Unidentifiable	53.4	100.0	83.3	74.6	82.7	69.2	14.2	37.4	10.78	31.5	36.4	14.7

*Note:* The name of each species has been shortened to smooth table formatting. *L. brau*, *L. brauni* (Chis), *L. longid*; *L. longidorsalis* (Chis), *L. caudo*; *L. caudovittatus* (Rub); *L. longif*., *L. longifilis* (Rub), *L. pauci*, *L. paucisquamatus* (Rub).

GLMs revealed a significant effect of the intestine length on insects and detritus –algae –moss proportions (in terms of volume) in *L. brauni* (Chi) with (*t* = −2.76, *p* < 0.01) for insects and a significant effect on the proportion of detritus –algae –moss with (*t* = 2.10, *p* < 0.05). A significant effect of intestine length was observed on detritus –algae –moss in *L. longifilis* (Rub) (*t* = 2.43, *p* < 0.05) (Supporting Information Table S1). Only the overall ANOVA model was significant for insects in *L. brauni* (Chi) (*F* value = 3.28, *p* = 0.03) (Supporting Information Table S1).

ANOSIM revealed an overall difference among diets (*r* = 0.35, *p* < 0.001) of the different species (Table S2). Pairwise ANOSIMs between species revealed that this overall difference was largely driven by *L. brauni* (Chi) having a significantly different diet compared to all other species, including the smiling phenotype (*r* = 0.41–0.63, *p* < 0.001), whereas *L. longidorsalis* (Chi), rubberlip species and the smiling phenotype had similar diets (*r* = 0.02–0.23, *p* > 0.05) (Supporting Information Table S2). Whenever pairwise differences were significant, SIMPER indicated that insects and detritus‐algae‐moss were the main food categories underpinning the observed dissimilarity between species (Table S2). Insects and detritus‐algae‐moss had 0.71 and 0.42 for *L. brauni* (Chi) and *L. longidorsalis* (Chi) respectively; 0.71 and 0.42 for *L. brauni* (Chi) and *L. longifilis* (Rub) respectively; 0.63 and 0.41 for *L. brauni* (Chi) and *L. paucisquamatus* (Rub) respectively; and 0.81 and 0.52 for *L. brauni* (Chi) and the smiling phenotype respectively.

### 3.3 Stable Isotope

#### 3.3.1 General Pattern of Isotopic Variability in Labeobarbus Species and Putative Food Sources within the Middle Luhoho

**FIGURE 4:**
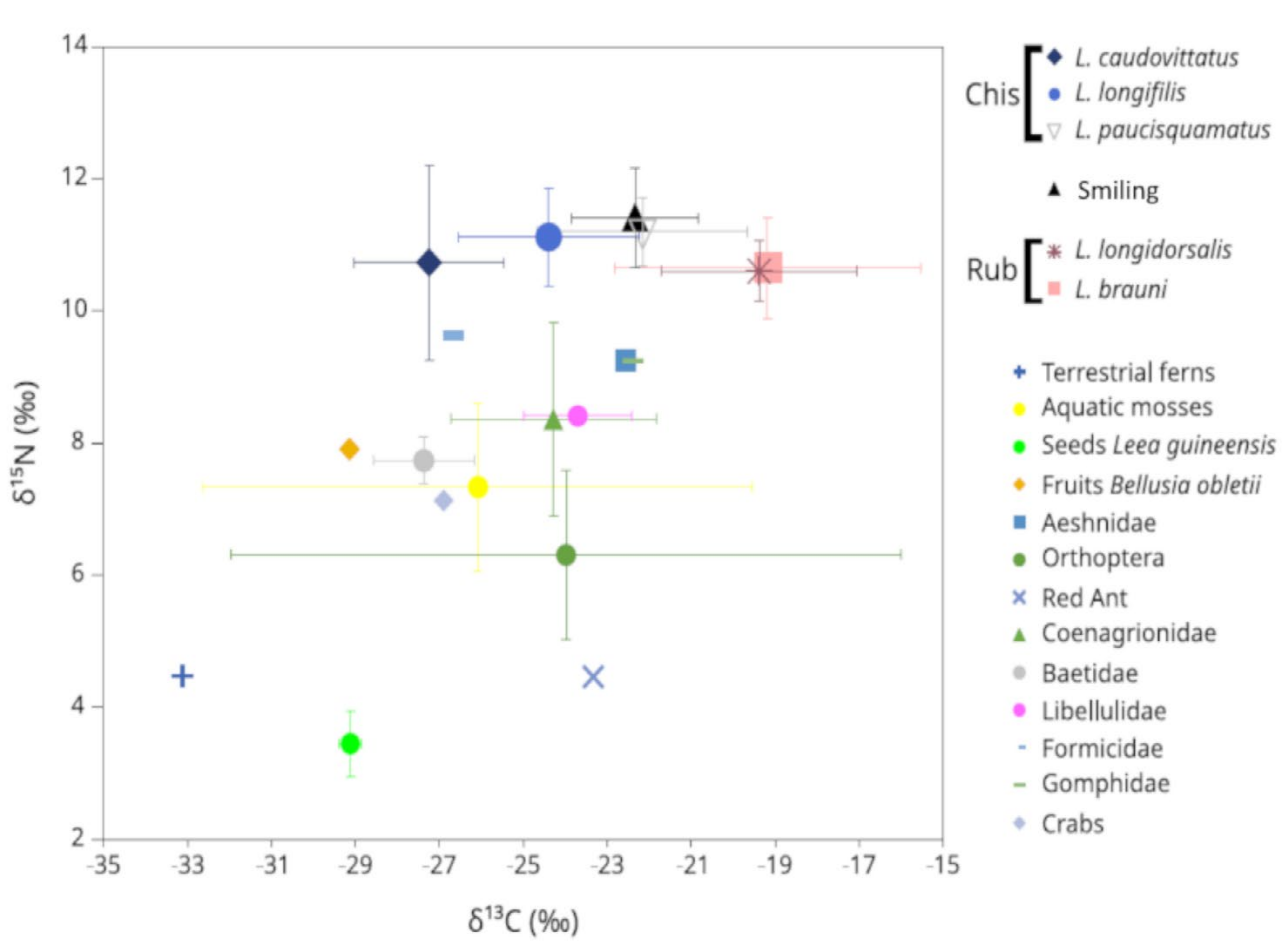
Biplot of mean δ^13^C and δ^15^N for chiselmouth (Chis) (*Labeobarbus brauni* and *L. longidorsalis*), rubberlip (Rub) (*L. caudovittatus*, *L. longifilis* and *L. paucisquamatus*), and smiling phenotypes (intermediate phenotypes), with putative preys. Whiskers represent the standard deviation.

At the overall scale (i.e., at all rivers), a pattern of isotopic differences among *Labeobarbus* phenotypes (chiselmouth vs. rubberlips vs. smiling phenotype) was observed. The observed difference mainly occurred along the carbon stable isotope (δ^13^C) axis, whereas all species including the smiling phenotype had similar δ^15^N values (Figure 4; Supporting Information Table S3). Carbon stable isotope (δ^13^C) values showed great variability, ranging between −28.7‰ and −13.6‰; with the most variable species being *L. brauni* (Chi) and, to some extent, *L. paucisquamatus* (Rub), whereas the smiling phenotype is intermediate. There was an overall pattern of C isotope disparity according to phenotype affiliation.

δ^13^C values (mean ± SD) were highest for the chiselmouth (−19.2‰± 3.2‰). Both species had almost the same δ^13^C values [−19.2‰ ± 3.6‰ for *L. brauni* (Chi) and −19.4‰± 2.2‰ for *L. longidorsalis* (Chi)]. Rubberlip species had the lowest δ^13^C values (−23.9‰± 2.4‰), with the lowest value observed in *L. caudovittatus* (Rub) (−27.2‰± 1.5‰), while *L. longifilis* (Rub) and *L. paucisquamatus* (Rub) values were respectively −24.4‰ ± 2.1‰ and 22.2‰ ± 2.4‰. The smiling phenotype had intermediate δ^13^C values (−22.3‰ ± 1.4‰), in between chiselmouth and rubberlip phenotypes (Figure 4; and Supporting Information Table S3). All pairwise tests between the δ^13^C values of chiselmouth and rubberlip species indicated significant differences (*p* < 0.05), while δ^13^C values were similar within each of the chiselmouth and rubberlip phenotypes (*p* > 0.05 in all cases) (Table 4). In addition, *L. paucisquamatus* (Rub) had significantly higher δ^13^C values compared to both rubberlip species (*p* < 0.05), whereas the smiling phenotype had significantly different δ^13^C values (intermediate) than the chiselmouth *L. longidorsalis* (Chi) (*p* < 0.05) and the rubberlip *L. caudovittatus* (Rub) (*p* < 0.05). Nitrogen stable isotope (δ^15^N) values ranged between 9.6‰ and 12.8‰ for all *Labeobarbus* species, but δ^15^N variation was not very pronounced within each species. δ^15^N values (mean ± SD) were 10.7‰± 0.7‰ for *L. brauni* (Chi) and 10.7‰ ± 0.4‰ for *L. longidorsalis* (Chi), 10.7‰± 1.3‰ for *L. caudovittatus* (Rub) and 11.1‰± 0.5‰ for *L. longifilis* (Rub), and 11.2‰± 0.5‰ for *L. paucisquamatus* (Rub) and 11.4‰± 0.7‰ for *L. paucisquamatus* (Rub) (Figure 4; Supporting Information Table S3). Overall, there were no significant differences in δ^15^N values among any species (Table 4). There was no clear pattern of isotopic variation with body size in any investigated species (data not shown).

**TABLE 4:** PERMANOVA pairwise tests of differences in isotopic composition (δ^13^C and δ^15^N) between pairs of *Labeobarbus* species from the middle Luhoho (global scale).

Pairwise tests	df	δ^13^C		δ^15^N	
		*t*	adj *p*	*t*	adj *p*
*L. brauni* (Chis), *L. caudovittatus* (Rub)	32	4.300	**0.002**	0.22	1.000
*L. brauni* (Chis), Smiling (Int)	39	2.760	0.105	2.83	0.117
*L. brauni* (Chis), *L. longidorsalis* (Chis)	38	0.150	1.000	0.21	1.000
*L. brauni* (Chis), *L. longifilis* (Rub)	62	7.03	**0.002**	2.550	0.195
*L. brauni* (Chis), *L. paucisquamatus* (Rub)	46	3.030	0.071	2.680	0.182
*L. caudovittatus* (Rub), Smiling (Int)	13	5.340	**0.015**	1.160	1.000
*L. caudovittatus* (Rub), *L. longidorsalis* (Chis)	12	6.110	**0.018**	0.310	1.000
*L. caudovittatus* (Rub), *L. longifilis* (Rub)	36	2.540	0.207	0.870	1.000
*L. caudovittatus* (Rub), *L. paucisquamatus* (Rub)	20	3.820	**0.020**	1.070	1.000
Smiling (Int), *L. longidorsalis* (Chis)	19	3.550	**0.049**	2.90	0.155
Smiling (Int), *L. longifilis* (Rub)	43	2.910	0.110	1.060	1.000
Smiling (Int), *L. paucisquamatus* (Rub)	27	0.230	1.000	0.890	1.000
*L. longidorsalis* (Chis), *L. longifilis* (Rub)	42	6.380	**0.002**	2.130	0.543
*L. longidorsalis* (Chis), *L. paucisquamatus* (Rub)	26	2.890	0.119	2.990	0.122
*L. longifilis* (Rub), *L. paucisquamatus* (Rub)	50	3.370	**0.026**	0.320	1.000

*Note:* ‘*t*’ = T statistics. ‘adj *p*’ = *p* values adjusted using the Bonferroni correction for multiple testing. Boldface indicates statistical significance (*p* < 0.05). Chis, Rub and Int refer to Chiselmouth, Rubberlip and intermediate mouth phenotypes, respectively.

The isotopic composition of putative food items (all sites confounded) globally showed an expected enrichment from primary producers to higher trophic levels in the aquatic trophic chain and showed high variation, particularly among terrestrial food types (seeds, fruits and terrestrial insects) (Figure 4). δ^13^C and δ^15^N values of a single sample of fern were −33.1‰ and 4.5‰, respectively. Aquatic mosses' δ^13^C and δ^15^N values were −26.1‰ ± 5.3‰ and 7.3‰ ± 1.0‰, respectively. δ^13^C and δ^15^N values were −26.1‰ (± 0.2‰) and 7.3‰ (± 1.0‰), −29.1‰ (± 0.2‰) and 3.4‰ (± 0.4‰), for aquatic mosses and the seed of *Leea guineensis*, Don, 1831, respectively; −29.1‰ and 7.9‰, respectively, for the fruit of *Bellucia aubletii*, commonly called ‘Manono’. All aquatic invertebrates were moderately enriched in stable isotopes. The mean or absolute value δ^13^C was −27.4‰ to 22.4‰. The mean or absolute value δ^15^N was 4.5‰ for Red Ant to 9.6‰ Ant.

We observed shifts in the isotopic composition of these species (mainly δ^13^C values) across the different rivers. δ^13^C values of *L. brauni* (Chi) were considerably lower in the Heke (between −26.4‰ and −18.3‰), highest in the Tchinganda (between −15.1‰ and 13.6‰), and intermediate in the Lwana (between −17.5‰ and −16.7‰; with *n* = 2) and Nyamunene (between −19.3‰ and −14.5‰). There was a similar pattern of isotopic variation for the two other species. For *L*. *longifilis* (Rub), δ^13^C values were lowest in the Heke (between −28.7‰ and −23.5‰), highest in the Luhoho (between −23.9‰ and −22.3‰) and intermediate in the Lwana (between −26.3‰ and −20.1‰). For *L. paucisquamatus* (Rub) δ^13^C values were lowest in the Heke (−27.8‰ with *n* = 1), highest in the Tchinganda (between −22.0‰ and  −17.9‰) and intermediate in the Nyamunene (between −26.6‰ and −20.3‰).

#### 3.3.2 Isotopic Niche Modelling in the Middle Luhoho

**FIGURE 5:**
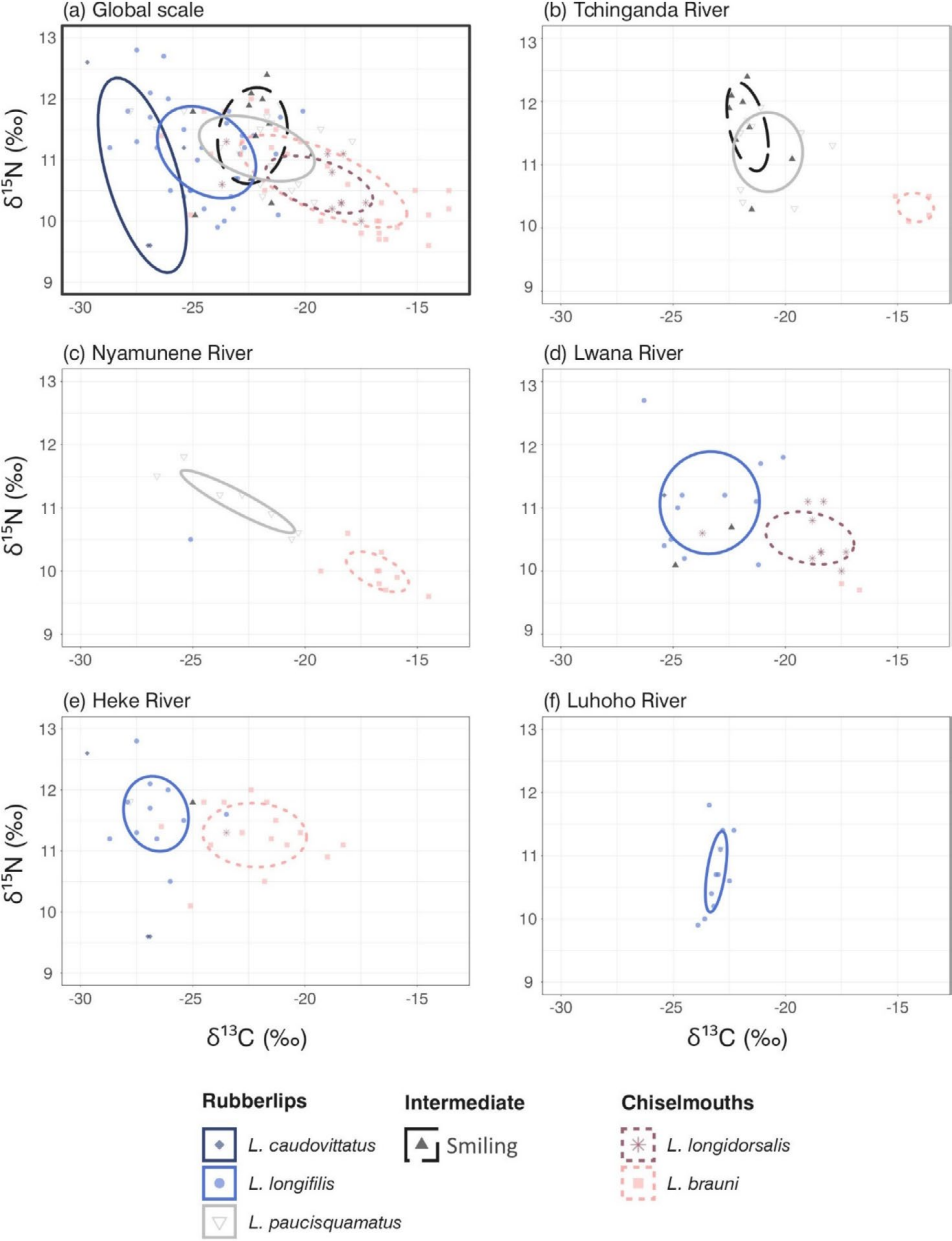
Isotopic niches (proxies for trophic niches) of *Labeobarbus* species for all sites combined (a: Global scale) and from the five sampled river stretches of the Middle Luhoho (b–f) in the space of carbon (δ^13^C) and nitrogen (δ^15^N) stable isotopes. Isotopic niches are represented as standard ellipses areas and encompass *ca*. 40% of one species isotopic variability. (a) Global scale (data from all river stretches combined), (b) Tchinganda River, (c) Nyamunene River, (d) Lwana River, (e) Heke River and (f) Luhoho River. *Labeobarbus longidorsalis* and *L. brauni* represent chiselmouth phenotype illustrated by dotted line, respectively in dark and light pink, *Labeobarbus caudovittatus*, *L. longifilis* and *L. paucisquamatus* belong to the rubberlip phenotype represented by solid line, in dark and light blue and dashed line in grey, respectively, and smiling phenotype is illustrated by dashed line in black.

Bayesian modelling of the isotopic niches provided congruent results. *Labeobarbus* species showed a conserved pattern of isotopic niche segregation among phenotypes (rubberlip vs. chiselmouth vs. smiling phenotypes) both at the general scale (data from all sites combined) and within each individual site (Figure 5). At the global scale (Figure 5a), both chiselmouth species occupied a very similar isotopic niche, with *L. longidorsalis* (Chi) niche located within the larger niche of *L. brauni* (Chi), corresponding to 50% of shared niche space between the two (Table 5). Similarly, isotopic niches of *L. paucisquamatus* (Rub) and smiling were very close [Shared niche space (SNS) = 48%], and largely different from those of both chiselmouth species [range of SNS = 2%–12%, except a slightly higher (SNS) = 24% between *L. paucisquamatus* (Rub) and *L. brauni* (Chi)] (Table 5). Rubberlip species had clearly lower δ^13^C values than all other *Labeobarbus*, but their isotopic composition also differed significantly among one another [SNS = 4% between *L. caudovittatus* (Rub) and *L. longifilis* (Rub)]. The niche of *L. caudovittatus* (Rub) did not overlap with that of chiselmouth nor with the other rubberlip species. Overlap between the niche of *L. longifilis* (Rub) and the chiselmouth was zero or very small [SNS = 0% and 2% with *L. brauni* (Chi) and *L. longidorsalis* (Chi), respectively], whereas *L. longifilis* (Rub) showed a low degree of overlap with smiling (SNS = 17) and 21% with *L. paucisquamatus* (Rub), respectively) (Table 5).

**TABLE 5:** Percentage of shared isotopic niche space between each pair of *Labeobarbus* species of the Middle Luhoho (global scale).

Percentage of shared isotopic niche space	*L. brauni*	*L. longidorsalis*	*L. caudovittatus*	*L. longifilis*	*L. paucisquamatus*
*L. longidorsalis*	50%				
*L. caudovittatus*	0%	0%			
*L. longifilis*	2%	0%	4%		
*L. paucisquamatus*	24%	12%	0%	21%	
Smiling	12%	2%	0%	17%	48%

There was no overlap of the species' niches belonging to different phenotypes (SNS = 0%) while studying populations in sympatry at the scale of individual river stretches. Despite apparent global shifts along the δ^13^C axis across different sites, the rubberlip *L. longifilis* (Rub) never overlapped with chiselmouth when found in sympatry [SNS = 0% with *L. brauni* (Chi) in Heke (Figure 5e)] and with *L. caudovittatus* (Rub) in Lwana [Figure 5d], and conserved a range of values within that of Lwana in Luhoho where it was found alone [Figure 5c]. Similarly, there was no overlap between *L. brauni* (Chi) and smiling phenotype when they occurred in sympatry, i.e., in Tchinganda and Nyamunene (SNS = 0% in both cases) (Figure 5e,f). In Tchinganda, *L. paucisquamatus* (Rub) and smiling occurred in sympatry and shared 48% of niche space (Figure 5f and Table 5).

In terms of isotopic niche width (SEA_B_), *L. brauni* (Chi) and *L. caudovittatus* (Rub) displayed wide isotopic niches [Mode (95% Credible Interval) = 6.1‰^2^ (4.3–8.9) and 6‰^2^ (2.2–22), respectively]; the latter being the most variable estimate, probably because of its low sample size. Both niches were similar in size (posterior probability of difference = 33%), whereas they were found to be larger than the five others with a posterior probability ranging between 84% and 97% (Supporting Information Table S4). *Labeobarbus longidorsalis* (Chi) depicted on average the smallest isotopic niche [2.6‰^2^ (1.4–5.4)], and was smaller than *L. brauni* (Chi) (probability = 97%) and the two rubberlip [*L. caudovittatus* (Rub), probability = 96%, and *L. longifilis* (Rub), 4.6‰^2^ (3.3–6.5), probability = 89%]. However, its size could not be differentiated from smiling and *L. paucisquamatus* (Rub), respectively 3.3‰^2^ (1.8–6.5) and 3.6‰^2^ (2.3–6.0) (Supporting Information Table S4).

## 4 Discussion

This study provided findings regarding the trophic ecology of multiple *Labeobarbus* species bearing different mouth phenotypes from the Middle Luhoho Basin (Upper Congo) using an integrative approach of gut morphometry, gut content and stable isotope analysis. The mouth phenotypes studied included two species with a keratinised cutting edge on the lower jaw (chiselmouth phenotypes), three species with a fleshy mental lobe (rubberlip phenotype) and a phenotype with an intermediate mouth morphology (without mental lobe on the lower jaw) called the smiling phenotype. All three approaches provided congruent results, showing a pattern of niche differentiation among *Labeobarbus* species belonging to different mouth phenotypes, both at the global scale and between sympatric species within individual river stretches. The results indicated that all *Labeobarbus* species, including the smiling phenotype, could be defined as generalist omnivores, but with chiselmouth, especially *L. brauni* (Chi), being more herbivorous on that spectrum, the rubberlips being more insectivorous, and the smiling phenotype having an intermediate trophic niche between chiselmouth and rubberlips.

Isotopic niche analysis revealed a consistent and full partitioning of niches between the different mouth phenotypes across different river stretches, except at one location between the rubberlip *L. paucisquamatus* (Rub) and the smiling phenotype. Both insects and plant materials (periphyton) were commonly encountered food items in the gut of all *Labeobarbus* species, while other food categories were less frequent and abundant. Chiselmouth indicated to be dependent on food from plant origin, whereas rubberlip species were revealed to be dependent on insects. This observation is congruent with findings published for other *Labeobarbus* species. In Lake Tana, *Labeobarbus* species harbour several feeding modes with diets including fish, detritus, molluscs and insects (Nagelkerke et al. 1994). Although fish prey was mainly documented in the gut contents of some species occurring in Lake Tana, insects were also substantial in the phenotypes ‘Lip’, ‘Intermedius’ and ‘Dark’ as seen on average 10%–20% of the diet in terms of volume and important in the phenotypes ‘Troutlike’ and ‘Big mouth‐Big eye’ as seen at 45% and 60%, respectively (Nagelkerke et al. 1994). Similar results were observed in river systems of Ethiopia where chiselmouth species were classified herbivorous–detritivorous feeders (Levin et al. 2021) and rubberlip species named generalised and lipped ectomorphs were found to be detritivorous and invertivorous feeders (Levin et al. 2019, 2023). These results together with our study suggest that having a chiselmouth and a developed mental lobe on the lower jaw (i.e., rubberlip phenotype) is associated with food from plant origin (detritus‐periphyton) and insect consumption respectively.

All food items were observed in all species regardless of their phenotype. Indeed, although both chiselmouth species *L. brauni* and *L. longidorsalis* relied on food items from plant origin, the gut content revealed that they also used insect food (FO 62.2% and 100% respectively). In contrast, *L. caudovittatus*, *L. longifilis*, *L. paucisquamatus* and smiling phenotype were revealed to be insectivorous, but the food items from plant origin were also present in their guts (13.7%–53.8% FO). An opportunistic or generalist feeding strategy could be evoked for *Labeobarbus* from the Middle Luhoho, which seems to be congruent with the ratio of intestine length over standard length. According to Matthes (1963), an intestine ratio of 1.0 is typical for carnivores, while a ratio of 3.5 is indicative for omnivores such as *L. altianalis* (Boulenger, 1900), another rubberlip species, and can be even higher in herbivorous species. As such, the intestine ratios of the studied *Labeobarbus* species (Table 2) suggest that they could all be qualified as omnivorous, such as in the non‐scapper form from Ethiopia (Levin et al. 2021, 2023). Chiselmouth, in contrast, are more herbivorous on that spectrum (see the results of δ^13^N lower in Tchinganda and Nyamunene where the species is syntopic). This is true, in particular, for *L. brauni* (Chi) which presented the highest intestine ratio (1.8–6.7) and to a lesser degree for *L. longidorsalis* (1.7–4.7). However, both have lower ratios compared to that of the scraping mouth phenotype from the Genale and Gojeb rivers in Ethiopia (ca. 2.5–7.0) (Levin et al. 2021). The higher intestine ratio of *L. brauni* (Chi) is also congruent with its low δ^15^N composition, which is used as a proxy for trophic level. The presence of insects in *L. brauni* and *L. longidorsalis* (see gut content approach but, not Stable Isotope Approach) attests to the opportunistic behaviour of *Labeobarbus* species in the Middle Luhoho. Many aquatic insect larvae could have been swallowed during stone scraping. A similar diet composition was found in *Acapoeta tanganicae* (Boulenger, 1900), a species with a similar lower jaw morphology whose diet comprised algae, epiphytic diatoms and aquatic insect larvae (Matthes 1963). *Labeobarbus brauni* (Chi) may feed on temporally, unusually abundant aquatic insects during their emergence (Gerking 1994), or occasionally consume fallback prey if their preferred resource becomes scarce (Paugy and Lévêque 2017). The occurrence of uncommon foods may rely on the propensity of *L. brauni* (Chi) to use a large scope of habitats in the Luhoho Basin (Kisekelwa et al. 2021). Indeed, a relation usually exists between habitat use and trophic ecology (Lévêque 1997).

The rubberlips would be more insectivorous, particularly *L. longifilis* (Rub) with the lowest ratio (Table 2) and Stable Isotope Analysis (SIA) (Figure 4). *Labeobarbus paucisquamatus* (Rub) and the smiling phenotype could be more omnivorous as they exhibited intermediate ratios, as seen in the smiling phenotype with 53.8% FO. Compared with findings on other *Labeobarbus* species, these ratios seemed to be lower than those found in *L. gananensis* (Vinciguerra, 1895) forms, which varied between *ca*. 4–6, except for the large‐mouthed form found in Genale (Levin et al. 2019). *Labeobarbus intermedius* (Rüppell, 1835), with an intermediate intestine ratio of *ca*. 3.2, is also omnivorous (Levin et al. 2021). Nevertheless, it was tricky to draw conclusions regarding the trophic niche of *Labeobarbus caudovittatus* (Rub) due to few available samples. Only four intestinal samples were collected from specimens that exhibit phenotypic variability in their lower jaw (Table 1). Two specimens have thick lips (rubberlips with a developed mental lobe) and have longer intestines, whereas the two others displayed a regular mental lobe and had shorter intestines (400–900 mm vs. 250–300 mm). The morphotype ‘thick lip’ is probably a new species (Kisekelwa 2019; Decru et al. 2022), confused with the morphotype with a regular mental lobe due to sharing a flexible last unbranched dorsal‐fin ray.

The advanced stage of prey decay due to digestion and/or crushing during ingestion prevented precise identification of most insect prey. Nevertheless, the trophic partition could be well discussed based on the composition of the food items between species. It was indicated that the isotopic niches were always fully separated between chiselmouth (lower δ^13^C values) and rubberlip species (higher δ^13^C values), whereas a partial overlap was detected within rubberlips as well as between rubberlip and the smiling phenotype at one location when *Labeobarbus* species were sampled in sympatry within a river (Figure 4b,d–f). Sympatric species may use the same category of resources but, at a finer scale, exploit different species to co‐exist (Gerking 1994). The lack of plant origin (periphyton) as they can hardly be sampled and sorted out (Marty and Planas 2008), impeded a clear interpretation of the food source for this species (Figure 4). For rubberlip species, probably the difference in isotopic niche derived from a difference in the distribution of insect prey items along the Middle Luhoho. Indeed, a quick sampling of insects in the surroundings of four sampled basins revealed a high diversity (Libellulidae, Gyrinidae, Notoneumouridae, Aeshnidae, Tipulidae, Coenagrionidae, Naucoridae, Perlidae, Baetidae, Hydrophilidae and Elmidae). The use of DNA‐based methods such as multi‐marker metabarcoding of gut contents can enable a more precise picture of the diet of omnivorous fish as this is a well‐established method to study fish trophic ecology, and it can offer unparalleled taxonomic resolution, allowing deciphering of cryptic trophic interactions within complex aquatic networks (Lejeune et al. 2022; Huyghe et al. 2023).

Shifts in the isotopic composition of *L. brauni* (Chi), *L. longifilis* (Rub) and *L. paucisquamatus* (Rub) along the δ^13^C axis across sites might be at least partly attributed to landscape differences and the distribution of prey resources in the river. The landscape of this area is represented by a transitional mountain forest that drains the Tchinganda and a relatively typical rainforest similar to which the Nyamunene, Lwana and Heke rivers drain (Kisekelwa et al. 2021). This riparian vegetation and agricultural practices along the catchment (C3 vs. C4 plants) can influence aquatic consumer food webs via terrestrial subsidies (Michener and Lajtha 2007). Terrestrial C3 plants, such as trees in this case, can influence aquatic food webs by providing subsidies such as plant materials and insects feeding thereupon, which are more depleted in ^13^C, denoted by low δ^13^C values. By contrast, due to their more efficient metabolism, C4 plants are typically more enriched in ^13^C, reflected by high δ^13^C values; therefore, influencing the aquatic food web by providing more enriched subsidies. Canopy cover can also reduce the importance of benthic primary production, which is enriched in ^13^C in aquatic food webs, by shading the river (Michener and Lajtha 2007; Allan and Castillo 2007), therefore shifting the community towards lower δ^13^C values. The natural vegetation along the catchment of the Heke and the Lwana is relatively preserved. In the Heke and Lwana rivers, isotopic niches are globally shifted towards more negative values, which might be linked to a potentially more preserved natural riparian vegetation (Kisekelwa et al. 2021). Indeed, both an increase in the availability of terrestrial subsidies and shading of benthic primary producers could lead to a more ^13^C depleted composition in consumers. By contrast, δ^13^C values are globally higher in the Tchinganda, which could potentially be influenced by more anthropogenic activities along its catchment.

A previous study has indicated that both *L. paucisquamatus* (Rub) and the smiling phenotype likely derived from hybridisation between *L. brauni* (Chi) and *L. longifilis* (Rub) (see Kisekelwa 2019). Interestingly, these two taxa are on the ground of trophic niche related, i.e., they occupy a similar but intermediate location in the isotopic space; between δ^13^C values of chiselmouth and the two other rubberlips. This suggests that in highly morphologically diverse lineages such as in *Labeobarbus*, hybridisation might fuel adaptive diversification by providing new phenotypes that promote niche differentiation (Kagawa and Takimoto 2018).

## Author Contributions

**Tchalondawa Kisekelwa:** conceptualisation (lead), data curation (lead), formal analysis (equal), investigation (lead), methodology (lead), project administration (lead), software (equal), validation (equal), writing – original draft (lead), writing – review and editing (equal). **Wilondja Alimasi:** data curation (supporting), investigation (supporting), writing – review and editing (supporting). **Mudagi** **Joyeuse:** investigation (supporting), writing – review and editing (supporting). **Musombwa Kubota:** investigation (supporting), writing – review and editing (supporting). **Heri Muzungu:** investigation (supporting), writing – review and editing (supporting). **Archimède Mushagalusa Mulega:** investigation (supporting), methodology (supporting). **Pieter Lemmens:** writing – review and editing (equal). **Emmanuel Vreven:** conceptualisation (supporting), funding acquisition (lead), investigation (supporting), writing – review and editing (equal). **Jos Snoeks:** conceptualisation (supporting), funding acquisition (supporting), investigation (supporting), supervision (lead), writing – review and editing (equal). **Mulungula Masilya:** formal analysis (supporting), writing – review and editing (equal). **Steven Bouillon:** methodology (supporting), writing – review and editing (equal). **Benjamin Lejeune:** formal analysis (equal), software (equal), validation (equal), writing – review and editing (equal).

## Conflicts of Interest

The authors declare no conflicts of interest.

## Supporting information


**Table S1.** The parameters of the GLMs performed on the insects and detritus‐algae‐moss proportions in the diet of *Labeobarbus* species. Explanatory variables are standard length of fish (SL), mouth width (MW) and intestine.

**Table S2.** ANOSIM parameters and average food items contribution to the dissimilarity obtained after computing analyses between the qualitative and quantitative composition of diet in *Labeobarbus* species in the Luhoho basin. Br ( *L. brauni* ), Logd (*L. longidorsalis*), Longf (*L. longifilis*), Pau (*L. paucisquamatus*), smil (Smiling).

**Table S3.** Range of stable isotope obtained for each *Labeobarbus* species/taxon.

**Table S4.** Pairwise comparisons of posterior distributions of standard ellipses area (SEA_B_) for each pair of *Labeobarbus* species from the Middle Luhoho (global scale). Percentages are direct probabilities that SEA_B_ of species × (column) is larger than SEA_B_ of species y (line).

## Data Availability

Current link: http://datadryad.org/stash/share/x9PGYM59onkIrlKosXeNYP0hQuGQVrtL5iQgyCBT0_g. The file containing data on standard length, mouth width, intestine length, gut contents and stable isotope composition of the studied *Labeobarbus* species has been uploaded to Dryad (https://doi.org/10.5061/dryad.qfttdz0s9).
