# Supplementary material for: Insight Into Trophic Niche Differentiation in Labeobarbus (Cyprinidae) in the Luhoho Basin (Upper Congo Basin)
Source: Ecol Evol. 2025 Apr 3;15(4):e71171. doi: 10.1002/ece3.71171 (PMC11968145; doi:10.1002/ece3.71171)
Supplement: Supplementary file 1 — Table S1. The parameters of the GLMs performed on the insects and detritus‐algae‐moss proportions in the diet of Labeobarbus species. Explanatory variables are standard length of fish (SL), mouth width (MW) and intestine. [file ECE3-15-e71171-s001.docx]

Supporting information Table S1. The parameters of the GLMs performed on the insects and detritus-algae-moss proportions in the diet of *Labeobarbus* species. Explanatory variables are standard length of fish (SL), mouth width (MW) and intestine.

|  | *Labeobarbus brauni* | | | |
| --- | --- | --- | --- | --- |
| **Insects** | Coefficients (Intercept) | Standard Error | t value | P |
| MW | 0.1 | 0.7 | 0.1 | 0.9 |
| SL | 0.5 | 0.9 | 0.5 | 0.6 |
| Intestine | −0.9 | 0.3 | −2.8 | **0.007**** |
| Anova test on the model |  |  | F value | Pr(>F) |
|  |  |  | 3. 3 | **0.027*** |
|  |  |  |  |  |
| **Detritus-algae-moss** | Coefficients (Intercept) | Standard Error | t value | Pr(>\|t\|) |
| MW | 0.9 | 0.9 | 1.0 | 0.3 |
| SL | −2.2 | 1.2 | −1.789 | 0.1 |
| Intestine | 0.9 | 0.4 | 2.1 | **0.040*** |
| Anova test on the model |  |  | F value | Pr(>F) |
|  |  |  | 2.308 | 0.086 |
|  |  |  |  |  |
|  | *L. longifilis* | | | |
| **Detritus-algae-moss** | Coefficients (Intercept) | Standard Error | t value | Pr(>\|t\|) |
| MW | −0.6 | 0.7 | −0.8 | 0.4 |
| SL | −0.8 | 0.8 | −1.1 | 0.3 |
| Intestine | 0.97 | 0.4 | 2.4 | **0.018*** |
| Anova test on the model |  |  | F value | Pr(>F) |
|  |  |  | F=2.3 | 0.081 |
