# Supplementary material for: Insight Into Trophic Niche Differentiation in Labeobarbus (Cyprinidae) in the Luhoho Basin (Upper Congo Basin)
Source: Ecol Evol. 2025 Apr 3;15(4):e71171. doi: 10.1002/ece3.71171 (PMC11968145; doi:10.1002/ece3.71171)
Supplement: Supplementary file 2 — Table S2. ANOSIM parameters and average food items contribution to the dissimilarity obtained after computing analyses between the qualitative and quantitative composition of diet in Labeobarbus species in the Luhoho basin. Br ( L. brauni ), Logd (L. longidorsalis), Longf (L. longifilis), Pau (L. paucisquamatus), smil (Smiling). [file ECE3-15-e71171-s003.docx]

Supporting information Table S2. ANOSIM parameters and average food items contribution to the dissimilarity obtained after computing analyses between the qualitative and quantitative composition of diet in *Labeobarbus* species in the Luhoho basin. Br (*L. brauni*), Logd (*L. longidorsalis*), Longf (*L. longifilis*), Pau (*L. paucisquamatus*), smil (Smiling).

|  | All species (i) | Br vs Logd (ii) | Br vs Longf (iii) | Br vs Pau (iv) | Br vs smil (v) | Logd vs Longf (vi) | Logd vs Pau (vii) | Logd vs smil (viii) | Longf vs pau (ix) | Longf vs smil (x) | Pau vs smil (xi) |
| --- | --- | --- | --- | --- | --- | --- | --- | --- | --- | --- | --- |
| ANOSIM parameters | | | | | | | | | | | |
| r | 0.347 | 0.419 | 0.426 | 0.626 | 0.414 | 0.020 | 0.147 | 0.018 | 0.065 | 0.035 | 0.229 |
| p | <0.001 | <0.001 | <0.001 | <0.001 | <0.001 | 0.551 | 0.551 | 0.347 | 0.082 | 0.310 | 0.003 |
| SIMPER outputs | | | | | | | | | | | |
| Insects |  | 0.713 | 0.711 | 0.629 | 0.807 |  |  |  |  |  | 0.605 |
| Detritus-algae-moss |  | 0.419 | 0.417 | 0.414 | 0.523 |  |  |  |  |  |  |
| Unidentified items |  |  |  | 0.830 |  |  |  |  |  |  | 0.790 |
