# Supplementary material for: Insight Into Trophic Niche Differentiation in Labeobarbus (Cyprinidae) in the Luhoho Basin (Upper Congo Basin)
Source: Ecol Evol. 2025 Apr 3;15(4):e71171. doi: 10.1002/ece3.71171 (PMC11968145; doi:10.1002/ece3.71171)
Supplement: Supplementary file 3 — Table S3. Range of stable isotope obtained for each Labeobarbus species/taxon. [file ECE3-15-e71171-s002.docx]

**Supporting information Table S3**. Range of stable isotope obtained for each *Labeobarbus* species/taxon.

|  | *L. brauni* | *L. longidorsalis* | *L. caudovittatus* | *L. longifilis* | *L. paucisquamatus* | Smiling phenotype |
| --- | --- | --- | --- | --- | --- | --- |
| δ^13^C | -26.4– -13.6 (12.8‰) | -23.7–-17.3 (6.4‰) | -29.7– -25.4 (5.3‰) | -28.7– -20.1 (8.6‰) | -27.8– -17.9 (9.9‰) | -25.0 – -19.7 (5.3‰) |
| δ^15^N | 9.6–12.0 (2.4‰) | 10.0–11.3 (1.3‰) | 9.6–12.6 (3‰) | 9.9–12.8 (2.9‰) | 10.3–11.9 (1.6‰) | 10.1–12.4 (2.3‰) |
