# Supplementary material for: Insight Into Trophic Niche Differentiation in Labeobarbus (Cyprinidae) in the Luhoho Basin (Upper Congo Basin)
Source: Ecol Evol. 2025 Apr 3;15(4):e71171. doi: 10.1002/ece3.71171 (PMC11968145; doi:10.1002/ece3.71171)
Supplement: Supplementary file 4 — Table S4. Pairwise comparisons of posterior distributions of standard ellipses area (SEAB) for each pair of Labeobarbus species from the Middle Luhoho (global scale). Percentages are direct probabilities that SEAB of species × (column) is larger than SEAB of species y (line). [file ECE3-15-e71171-s004.docx]

Supporting information Table S4. Pairwise comparisons of posterior distributions of standard ellipses area (SEA_B_) for each pair of *Labeobarbus* species from the Middle Luhoho (global scale). Percentages are direct probabilities that SEA_B_ of species x (column) is larger than SEA_B_ of species y (line).

| Pairwise comparison: SEA_B_ x > SEA_B_ y | *L. brauni* | *L. longidorsalis* | *L. caudovittatus* | *L. longifilis* | *L. paucisquamatus* |
| --- | --- | --- | --- | --- | --- |
| *L. longidorsalis* | **97%** |  |  |  |  |
| *L. caudovittatus* | 33% | **96%** |  |  |  |
| *L. longifilis* | 87% | 11% | 84% |  |  |
| *L. paucisquamatus* | 94% | 26% | 91% | 77% |  |
| Smiling | 93% | 67% | 91% | 22% | 44% |
